# Effects of methane ratio on MPDF (micro-pilot dual-fuel) combustion characteristic in a heavy-duty single cylinder engine

**DOI:** 10.1038/s41598-021-89161-z

**Published:** 2021-05-07

**Authors:** Minhoo Choi, Khawar Mohiuddin, Sungwook Park

**Affiliations:** 1 Graduate School of Hanyang University, 222 Wangsimni-ro, Seongdong-gu, Seoul, 04763 Republic of Korea; 2 School of Mechanical Engineering, Hanyang University, 222 Wangsimni-ro, Seongdong-gu, Seoul, 04763 Republic of Korea

**Keywords:** Mechanical engineering, Diesel fuel

## Abstract

In this study, the characteristics of micro-pilot dual-fuel combustion with respect to the fuel mixture ratio in a single cylinder dual-fuel engine have been investigated. In order to analyze the characteristics of micro-pilot dual-fuel combustion, a metal engine and an optical single cylinder dual-fuel engine were used. The fuel mixture ratio was varied for experimental purposes; the diesel was directly injected into combustion chamber and the methane gas was supplied via intake port. The present study reports that increasing the methane mixture ratio from 0 to 97.67% changes the diesel combustion to pre-mixed combustion. As a result, the peak cylinder pressure was increased from 184 to 198 bar, and the rate of heat release was greatly advanced. In the MPDF condition, the nitrogen oxides emissions were reduced by about 90%p, and the fuel conversion efficiency increased about 5%p because of the low combustion temperature of pre-mixed combustion. However, for the same reason, the hydrocarbon emissions were increased about 95%p. The fastest combustion speed was found form the results of methane mixture ratio between 40 and 80%. In the condition of diesel combustion and micro-pilot dual-fuel combustion, the combustion periods of middle and initial were increased, respectively, resulting in the low combustion speed. The standard deviation of peak cylinder pressure, which represents the combustion variation, was correlated with initial combustion period. While the condition of methane gas mixture ratio between 40 and 80% shows the lowest combustion variation, the highest combustion variation was occurred by MPDF condition. Through the optical engine experiment, it can be found that the cycle to cycle combustion variation is ascribed to the turbulent flow and the variation of ignition position. The combustion images show that the unpredictable characteristics of the ignition position and slow flame propagation speed caused the combustion variation in micro-pilot dual-fuel combustion.

## Introduction

Heavy-duty CI engines are well known as the power generators most often used in transportation and heavy industry. Though diesel fuel has traditionally been used for heavy-duty CI engines, nowadays it has been implicated as a source of environmental problems. The diesel fuel which directly injected into the combustion chamber forms a stoichiometric and locally rich mixture with locally rich areas, and each of two different regions are the main sources of NOx and particulate matter (PM) emissions, respectively^1,2^. While the region of stoichiometric mixture which makes the high combustion temperature increases the NOx emissions, the PM emissions are increased by decreasing the oxygen utilization due to deteriorating homogeneity of the mixture in the combustion chamber^1,2^. In addition, although there are various reasons for high carbon dioxide emissions in diesel engine, the primary reason is that diesel fuel has more carbons compared with other fuels, which are converted to carbon dioxide during the combustion process. ^3^ For this and other reasons, it is hard to reduce the NOx, PM and CO_2_ emissions while using diesel, and it has been through efforts to install turbochargers or after treatment devices that recently strengthened emissions regulations have been met^4–6^. These solutions have, however, caused increased engine cost, and cannot serve as a fundamental solution for reducing emissions.

To reduce the environmental problems associated with diesel emissions, efforts have been applied to explore alternative fuels and to establish an improved combustion strategy. Among the work of many studies, this study has focused on dual-fuel combustion for alternative fuel and combustion strategies. According to previous studies, dual-fuel combustion can reduce NOx, PM and CO_2_ emissions compared with diesel combustion^7–12^. Kim et al.^7^ provided that the NOx and PM emissions were reduced about 50% under the condition of pre-mixed ratio 90%. However, such a high pre-mixed ratio increased the incomplete combustion emissions HC and CO about 100% compared to the reference conditions. At the same time, the unstable combustion caused by pre-mixed combustion increased the combustion variation. Serrano et al.^8^ analyzed the effects of high methane gas ratio 97% on the combustion characteristic in a 4-cylinder 1.6 L engine. They report that regardless of the engine load, the CO_2_ emissions of dual-fuel condition were always lower than that of diesel 100% condition. As the engine load increases, the difference of CO_2_ emissions with respect to the fuel mixture ratio was increased from 12 to 28%. The method for supplying the fuel into combustion chamber was determined by the fuel characteristics^13,14^. Generally, The dual-fuel combustion uses the liquid or pre-mixed gas fuel, which has different properties^13^. Ahmad et al.^13^ conducted the optical engine experiment by using the diesel and methane gas. They supplied methane gas with the intake air into the combustion chamber through an intake port, and the diesel was directly injected by an injector which installed in the combustion chamber. This study shows that as decreasing the diesel mixture ratio from 28 to 16%, the ignition delay was increased about 1 ms. This result is ascribed to the using the two different reactivity fuels in DF combustion. As above mentioned, the high reactive fuel was directly injected into combustion chamber during the compression stroke. And the high reactive fuel is the ignition source of the low reactivity and primary fuel which was supplied via intake port and mixed with air. The mixture was ignited by ignition source and pre-mixed combustion occurred. In order to prevent increased knocking combustion and NOx emissions, diesel fuel was preferred to direct injected fuel with characteristics of high cetane number with short ignition delay^13,15^. Yoshimoto et al.^15^ analyzed the effects of cetane number on combustion and emissions characteristics in a dual-fuel engine. Based on the natural gas, the heptamethylnonane, gasoline and n-hexadecane with cetane number 105, 60.2 and 100 were used as an ignition source, respectively. This study reported that n-hexadecane, which has the highest cetane number, decreases the ignition delay about 2 degree compared to the other fuels, resulting in the least misfiring by reducing the combustion variation. Unlike the direct injected fuel, the pre-mixed fuel of high octane number combined with long ignition delay was preferred for port injection fuel^13,16^. The methane gas is the most popular pre-mixed gas fuel used in dual-fuel engines and is the cleanest fuel with the lowest PM emissions due to its highest hydrogen-carbon ratio. The methane gas can significantly reduce PM emissions, as well as provide stable combustion with high octane number fuel while conferring anti-knocking properties^12,16,17^. For these reasons, this study used the methane gas as a main source of power. The dual-fuel combustion characteristics are determined by diesel and pre-mixed gas mixture ratio. Many studies have therefore been conducted for analyzing the effects of fuel mixture ratio on the characteristics of dual-fuel combustion^9,18^. Wang et al.^18^ conducted the dual-fuel PCCI combustion experiment under different dimethyl ether and diesel mixture ratio. They increased DME mixture ratio from 0 to 40%. Regardless of operating conditions, increasing the DME mixture ratio not only advanced the mass fraction burned (MFB) crank angle (CA) 50 more than 4 degree, but also reduced the NOx and smoke emissions about 10% and 50%, respectively. Sahoo et al. ^9^ conducted a gas and diesel dual-fuel combustion experiment with different fuel mixture ratio. Experiment results show that the dual-fuel combustion mode decreases the peak cylinder pressure about 5% compared to the diesel combustion mode. However, the pressure gradient of dual-fuel combustion is always higher than that of diesel combustion. Decreasing diesel mixture ratio changes the diesel combustion to pre-mixed combustion, resulting in the high combustion variation^19–22^. Duraisamy et al.^21^ analyzed the effects of EGR rate and pre-mixed gas ratio on the combustion variation of methanol and diesel dual-fuel reactivity controlled compression ignition (RCCI) combustion. This study found that pre-mixed methanol fuel mixture ratio increased the combustion variation. Under the middle engine load, the cyclic variation of indicated mean effective pressure (IMEP) was increased from 0.79 to 2.79% by increasing the methanol mixture ratio 90%. In addition, Cinar et al. ^22^ investigated the effects of fuel mixture ratio on emissions and the combustion variation by using the single cylinder diesel engine. They used diethyl ether (DEE) and diesel fuel for dual-fuels and found that the maximum cylinder pressure and pressure gradient was proportional with DEE mixture ratio which caused ignition delay. While the combustion variation was small in diesel combustion mode, the exceeding 20% pre-mixed DEE ratio led to increase the variation of IMEP and peak cylinder pressure, which are correlated with the knocking combustion intensity. Since the combustion variation deteriorates the engine performances and durability, it is the most important problem to be solved in the pre-mixed combustion type engine^23^.

Although a lot of researches have been conducted on the dual-fuel combustion engine, few studies have been investigated the characteristics of micro-pilot dual-fuel (MPDF) combustion. In particular, the fundamental study for analyzing the high combustion variation of MPDF combustion was hardly performed. Therefore, the present study focused on the characteristics of MPDF combustion with respect to the fuel mixture ratio in a heavy duty dual-fuel engine. For this study, the metal and optical engine experiments were performed with the different fuel mixture ratio. The effects of fuel mixture ratio on the combustion phase, energy balances, emissions and combustion duration were investigated.

## Experiment method

In order to investigate the characteristics of MPDF combustion which affected by the diesel and methane mixture ratio, engine experiments were conducted with various fuel mixture ratio.

### Metal engine experiment apparatus

**Figure 1:**
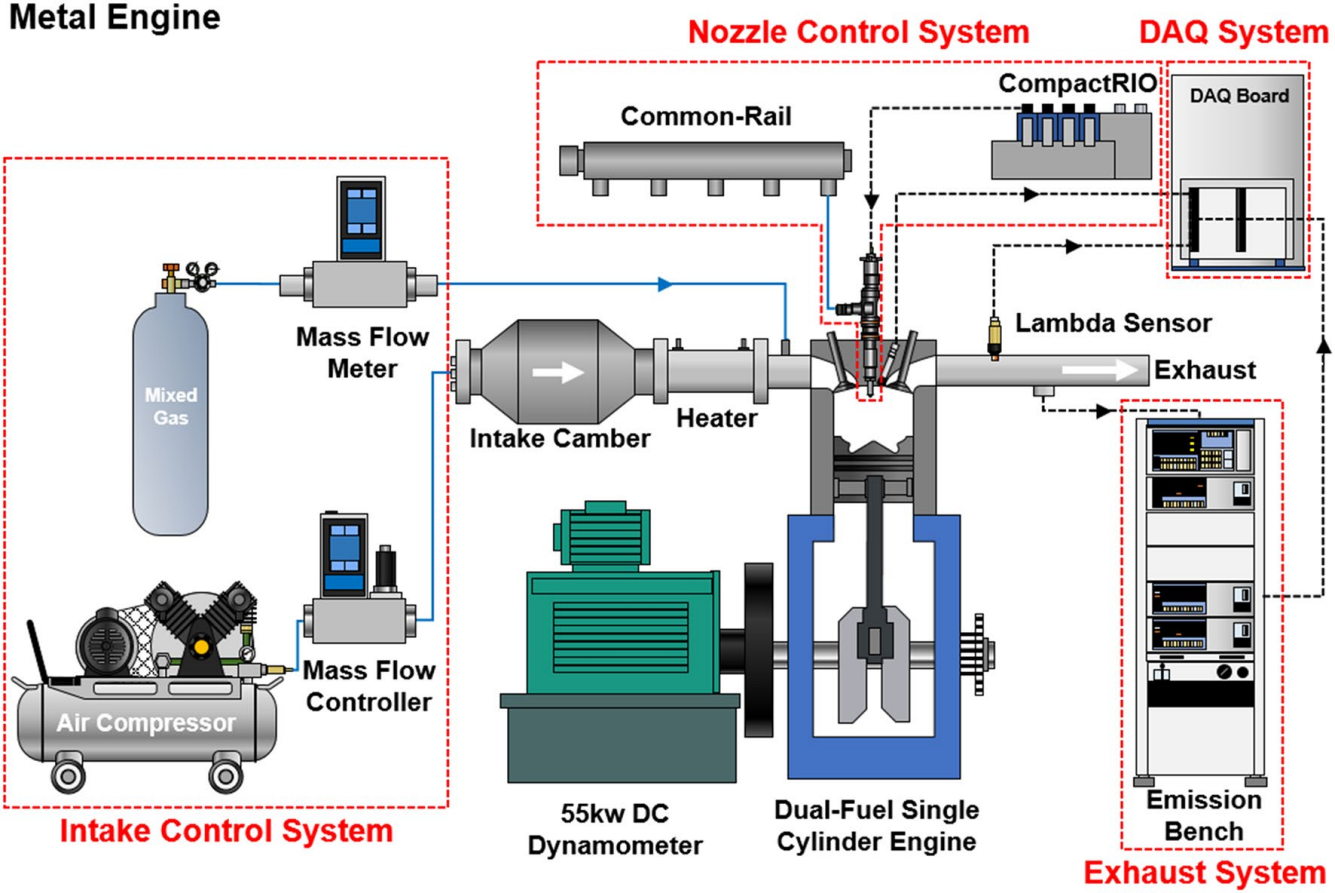
Schematic diagram of experimental metal engine.

The single cylinder dual-fuel engine used for this study consists of a metal engine and an optical engine. The Table 1 summarized the specification of single cylinder dual-fuel engine. The conventional single cylinder engine was used to conduct the experiment in high load operating conditions with combustion pressure of more than 200 bar. The metal engine consists of four groups: intake control system, engine control system, data acquisition system and an emissions of gases and smoke acquisition system. The schematic of the metal engine experimental setup is given in Fig. 1. In order to keep the air flow constant during engine experiments, an air compressor was used, and the air flow rate was controlled by a mass flow controller (MFC). The pre-mixed gas line was installed at the intake ports and flow rate was controlled by the manual valve in front of mass flow meter (MFM). The common rail was used for supplying diesel fuel under high injection pressure conditions and the diesel injection timing and injection duration were controlled by NI-LabVIEW system. Prior to conduct the experiment, diesel flow rate was tested to determine the injection durations that correlate with the fuel mixture ratio. The exhaust system was used to obtain emissions gas data during the experiment. A MEXA HORIBA 9100D exhaust analyzer was connected with exhaust port and measured emissions gas components. NI-DAQ Board and NI LabVIEW program comprised the DAQ system supporting data acquisition and post processing. In order to obtain reliable experimental data, the experiment was conducted under the stabilized states, and 100 cycles of experimental data were averaged by post processing.

**Table 1:** Specification of single cylinder dual-fuel engine.

Engine parameters	Specification
Bore (mm)	107
Stroke (mm)	126
Connecting rod (mm)	200
Displacement volume (mm^3^)	1100
Compression ratio	17.0
Number of injector nozzle holes	7
Nozzle hole diameter (mm)	0.174

### Metal engine experiment conditions

The reference metal engine experimental conditions of this study are given in the Table 2. In the condition of MPDF combustion, inappropriate injection timing and injection pressure causes the misfiring or the knocking combustion. Prior to the experiment, the optimized injection pressure 500 bar and diesel injection timing bTDC 27 degree to prevent the misfiring and knocking combustion are determined. In this study, the diesel and methane gas are used. The diesel fuel is liquid phase and the methane is gas phase. The details of fuel mixture conditions are shown in Table 3. The summation of low heating value of fuel was fixed to 3439.36 J/cycle. Based on the low heating value and fuel mixture ratio, diesel injection quantities and the methane gas flow rate were calculated, respectively. The condition of the highest methane mixture ratio was defined as the MPDF condition. To determine the minimum quantity of diesel injection required for stable MPDF combustion, diesel injection quantity was gradually increased until the misfiring no longer occurred. The minimum diesel injection quantity required for MPDF combustion was demonstrated at 1.33% of total fuel energy.

**Table 2:** The reference metal engine experimental conditions.

Reference conditions	Description
Engine speed (RPM)	900
Intake temperature (℃)	35
Intake pressure (bar)	1.95
Exhaust pressure (bar)	1.00
Water temperature (℃)	80.0
Oil temperature (℃)	80.0
Injection pressure (bar)	500
Diesel injection timing (aTDC, degree)	− 27

**Table 3:** The metal engine varied fuel mixture experimental conditions.

CH_4_ gas (%)	CH_4_ gas (J/stroke)	Diesel (%)	Diesel (J/stroke)
0.0	0	100.0	3439.36
20.0	687.87	80.0	2751.49
40.0	1375.74	60.0	2063.61
60.0	2063.61	40.0	1375.74
80.0	2751.49	20.0	687.87
85.0	2923.46	15.0	515.90
90.0	3095.42	10.0	343.94
95.0	3267.39	5.0	171.97
98.67	3393.53	1.33	45.83

### Optical engine experiment apparatus

**Figure 2:**
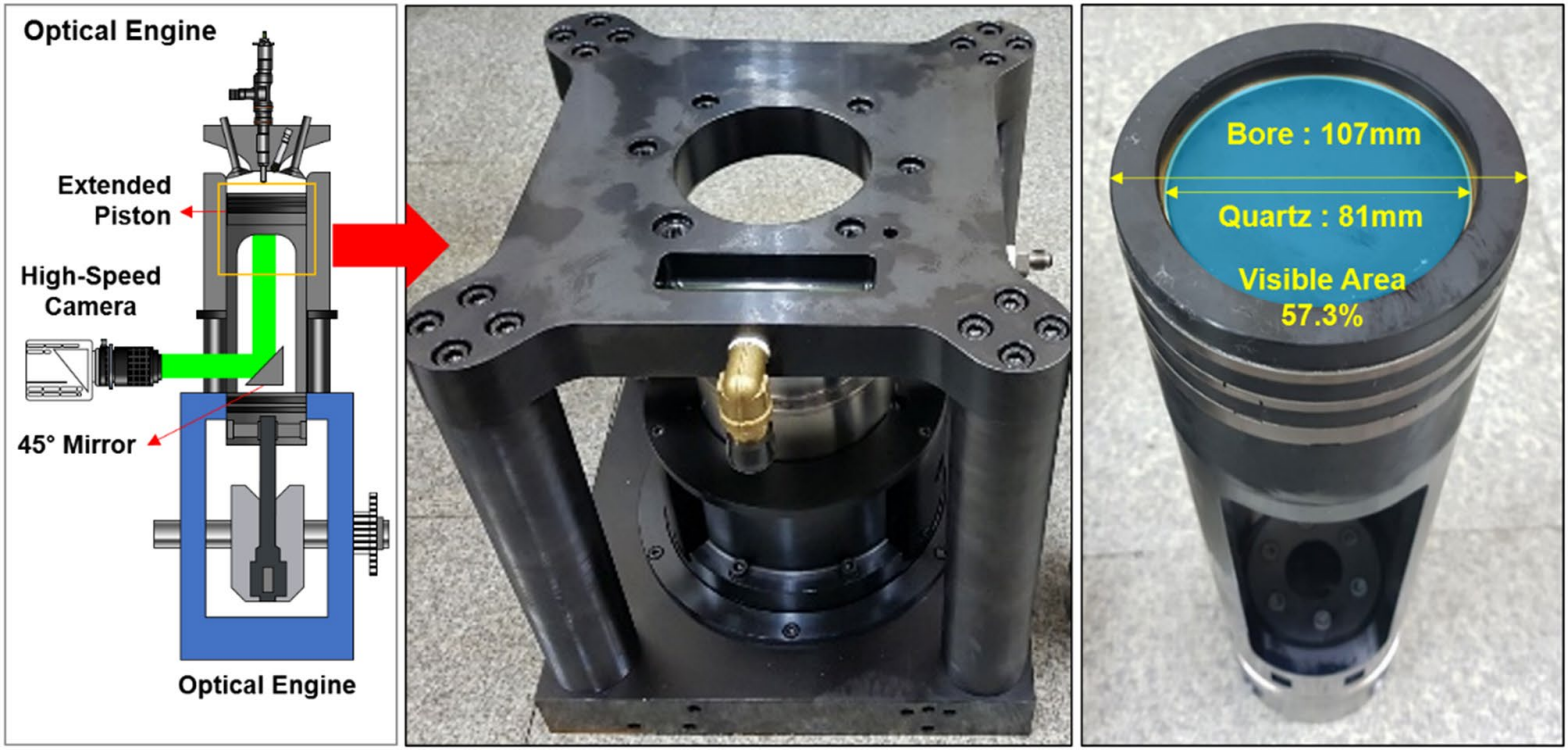
Schematic diagram of experimental optical engine.

Figure 2 illustrates the schematic of optical engine experimental setup. The optical engine has the same compression ratio as the metal engine, but optical was designed to acquire the bottom view image by adding an extension piston between the engine head and the crank case, which are in the same location in the metal engine. The extension piston is equipped with a flat transparent quartz piston which allows observation of the inside of the combustion chamber during the combustion, and the visualization area covers 57.3% of the total area of the combustion chamber. In addition, a 45 degree angle mirror was installed at the low part of the extension piston to obtain a flame image from the front of the engine. Phantom high speed camera was used to acquire flame image. The camera speed was adjusted to the engine speed to capture one image during crank angle of 1 degree. The injector signal was converted into a digital signal and it was used as a trigger for operating the high speed camera.

### Optical engine experiment conditions

Unlike the metal engine, the optical engine was used to conduct the experiment in low load operating conditions. In consideration of experimental safety and the possibility of damage to the quartz piston under high cylinder pressure, it was hard to match the optical experimental conditions with that of the metal engine. Therefore, the optical engine experiment is conducted under the lower engine load by reducing the intake air and pre-mixed gas flow rate. The details of experimental conditions for the optical engine are shown in the Tables 4 and 5. The diesel injection timing was retarded to obtain the same ignition delay as the metal engine. However, if the diesel injection quantity is reduced at the same rate as the reduction of total fuel energy, it is hard to ignite methane gas and the combustion variation can be increased. Therefore, the diesel injection quantity for MPDF combustion was the same as for the metal engine. If the diesel mixture ratio increased by more than 15%, the flame propagation speed was too fast making it difficult to analyze the difference between changes in the fuel mixture ratio as the flame propagated outside the visible area. Moreover, in consideration of the increasing pressure gradient which could be a problem with experimental safety as diesel mixture ratio increased. In the optical engine experiment, the diesel mixture ratio was limited to 15% of the low heating value of diesel and methane gas.

**Table 4:** The reference optical engine experimental conditions.

Reference conditions	Description
Engine speed (RPM)	900
Intake temperature (℃)	35
Intake pressure (bar)	1.24
Exhaust pressure (bar)	1.00
Water temperature (℃)	80.0
Oil temperature (℃)	80.0
Injection pressure (bar)	500
Diesel injection timing (aTDC, degree)	− 11

**Table 5:** The optical engine experimental condition of fuel mixture ratio.

CH_4_ gas (%)	CH_4_ gas (J/stroke)	Diesel (%)	Diesel (J/stroke)
85.0	1821.70	15.0	321.48
87.5	1875.28	12.5	267.90
90.0	1928.86	10	214.32
92.5	1982.44	7.5	160.74
95.0	2036.02	5	107.16
97.86	2097.35	2.14	45.83

## Results and discussion

### Metal engine experiment for the effects of fuel mixture ratio

**Figure 3:**
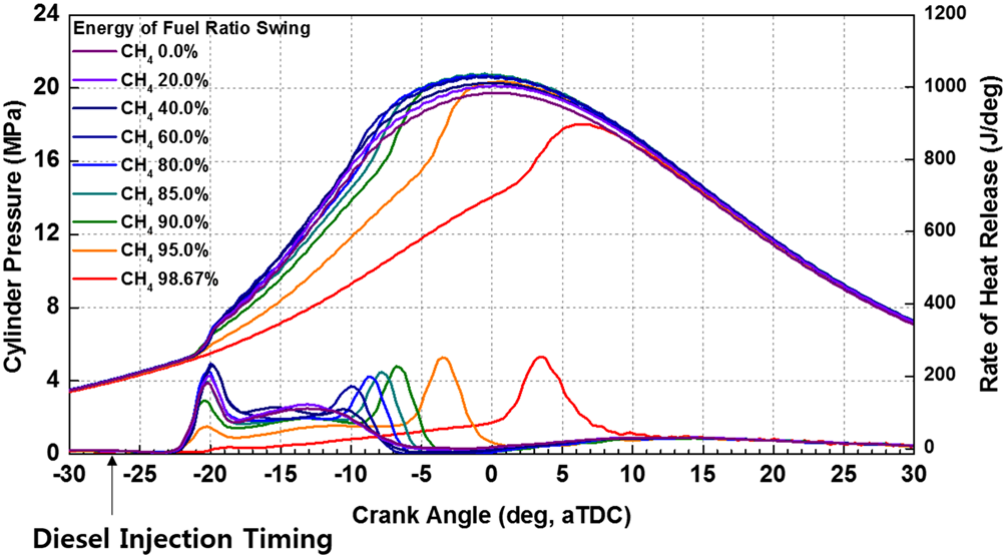
Cylinder pressure and ROHR with respect to the fuel mixture ratio.

**Figure 4:**
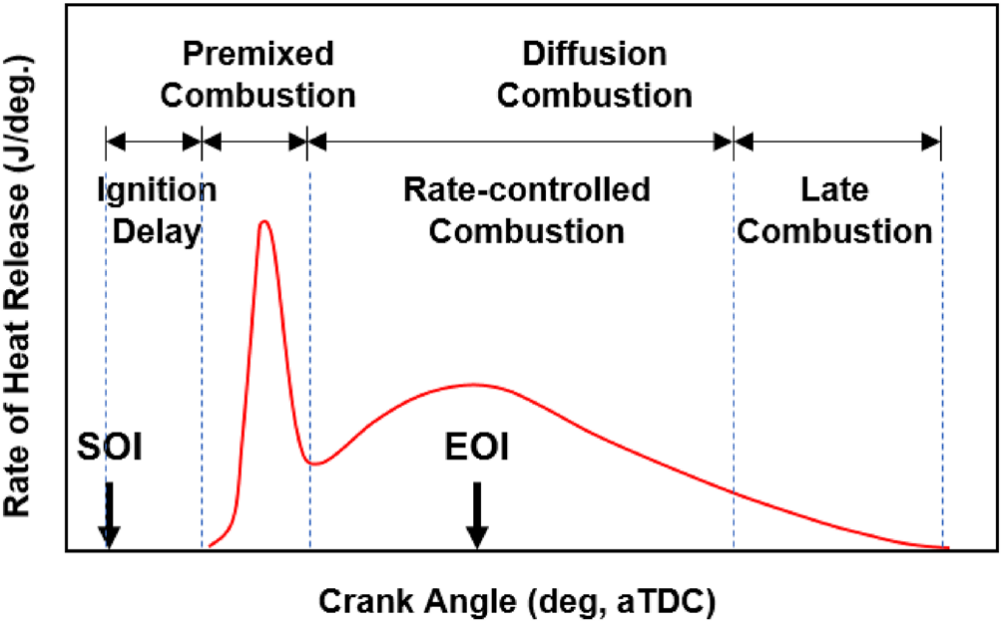
Combustion phase of diesel fuel ^33^.

**Figure 5:**
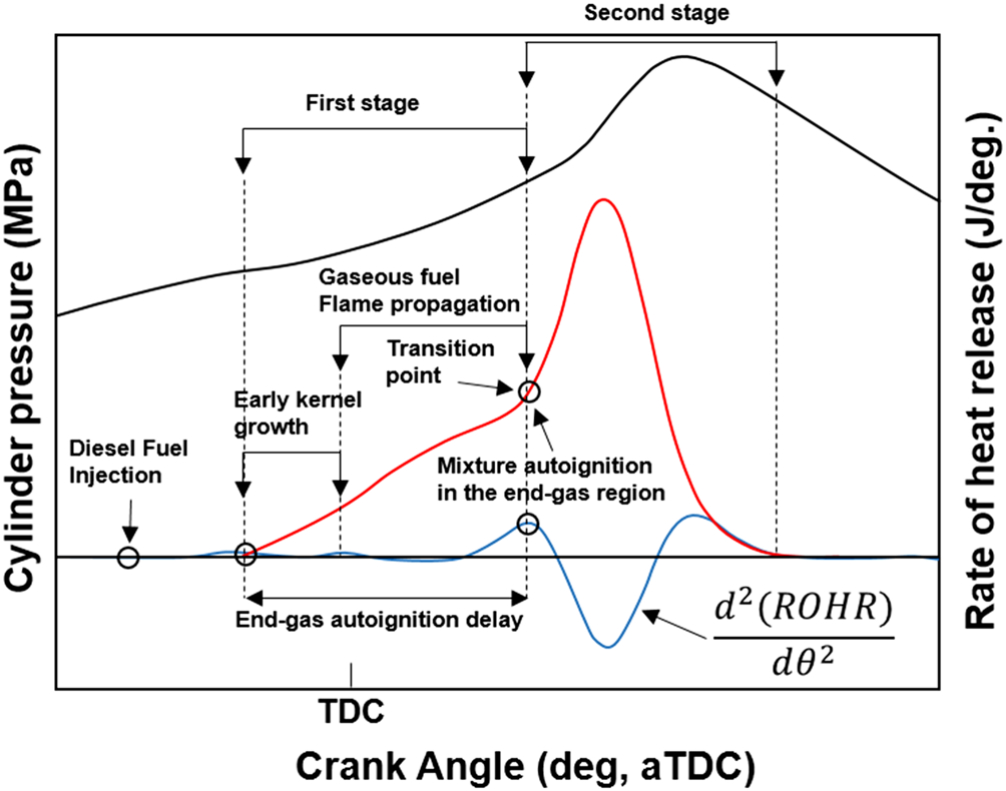
PREMIER combustion phase of MPDF ^26^.

**Figure 6:**
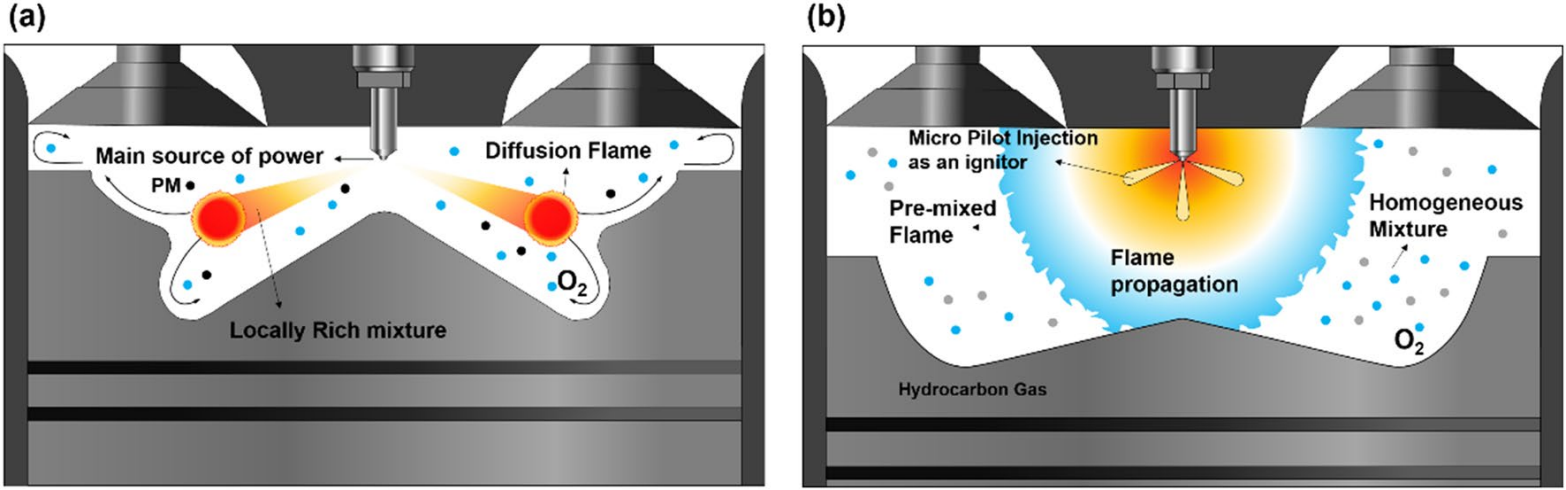
Comparison the combustion mechanism of **(a)** diesel combustion and **(b)** MPDF combustion.

**Figure 7:**
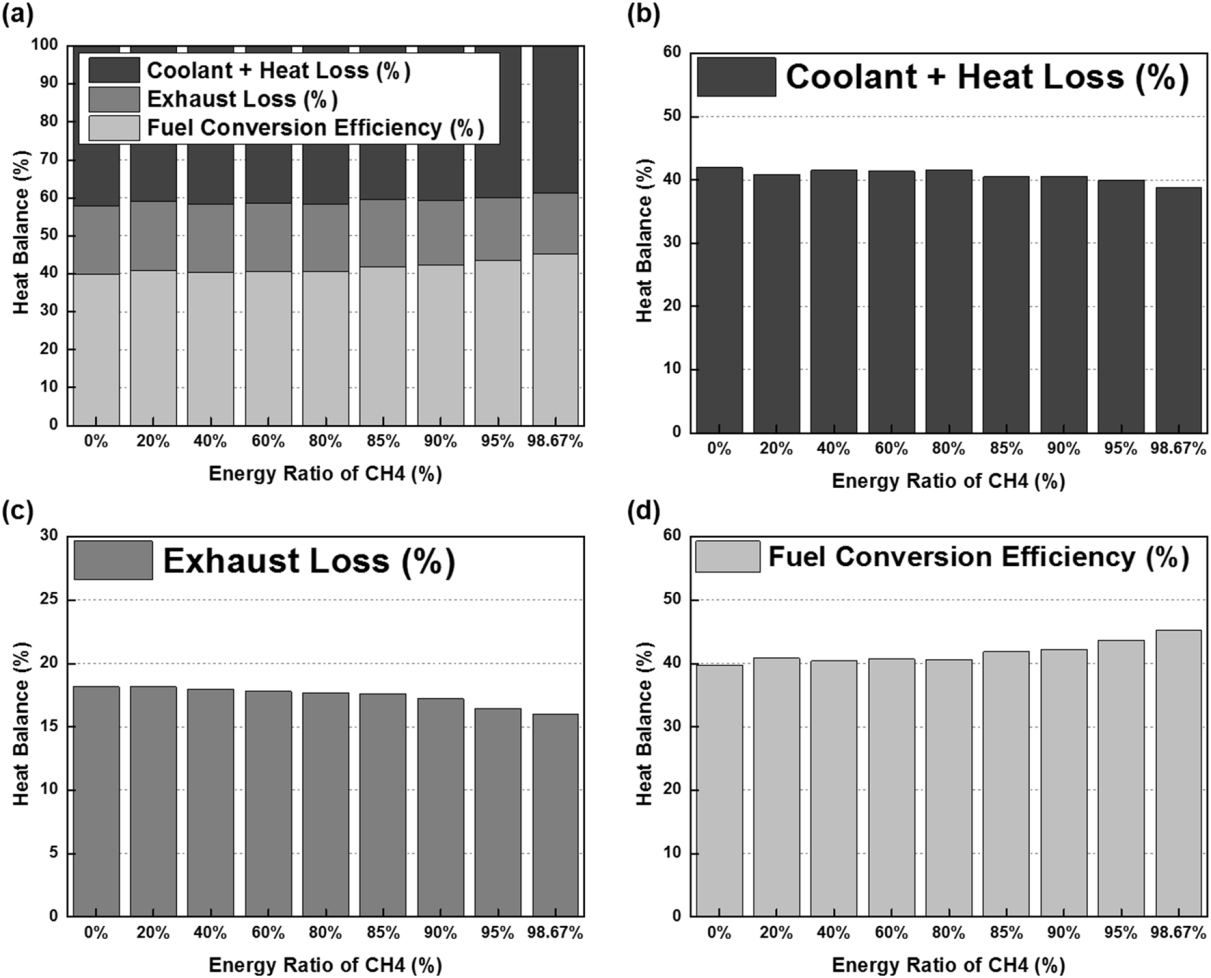
Energy balances with respect to the fuel mixture ratio.

**Figure 8:**
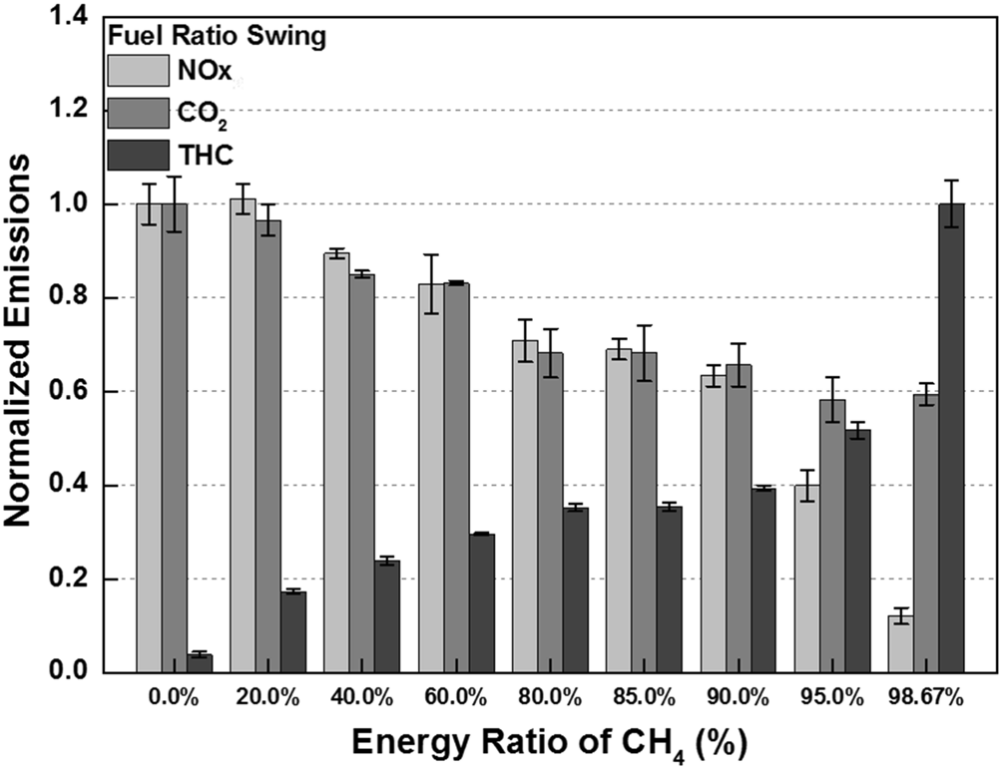
Emission gases with respect to the fuel mixture ratio.

**Figure 9:**
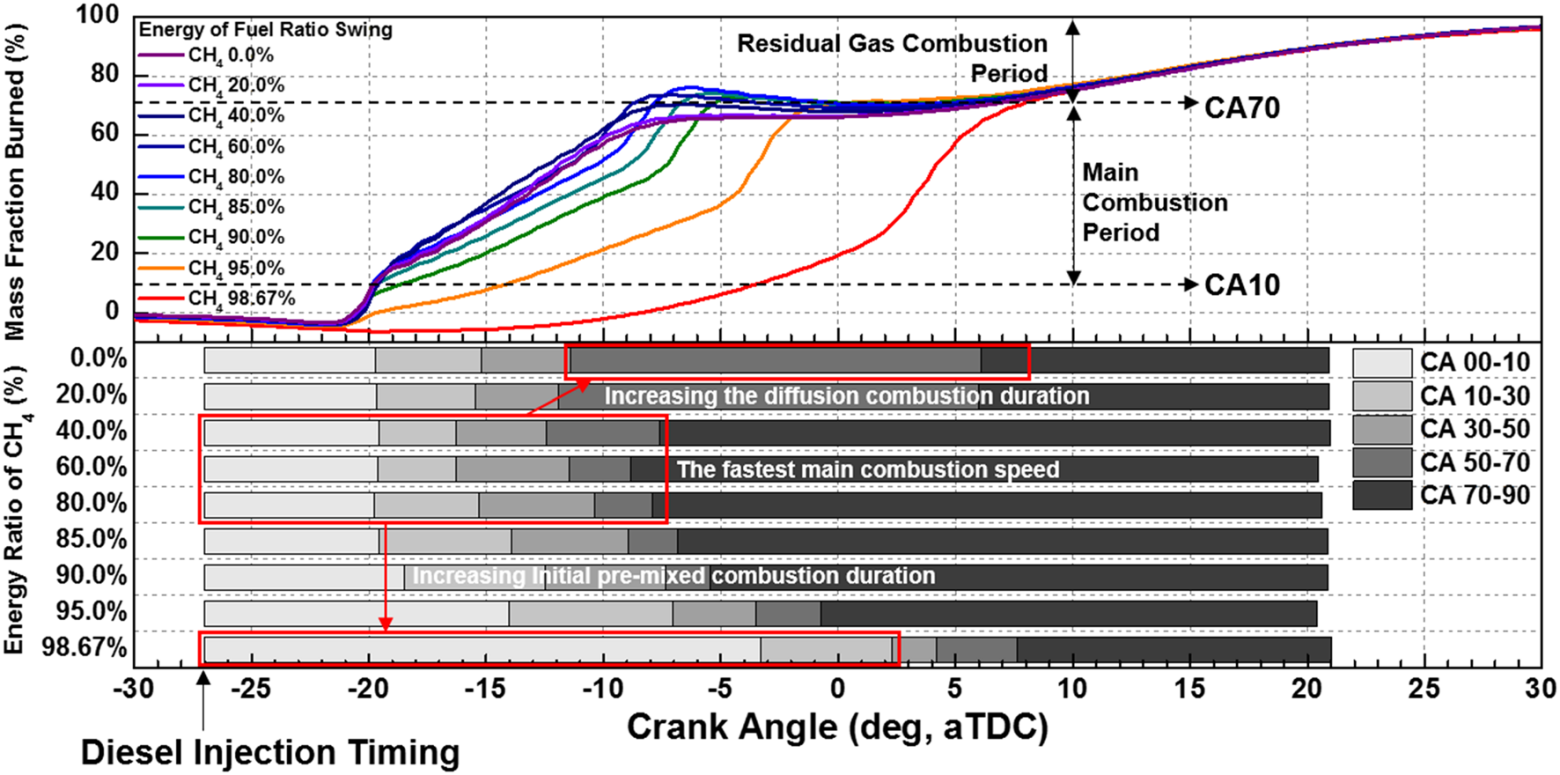
MFB with respect to the fuel mixture ratio.

**Figure 10:**
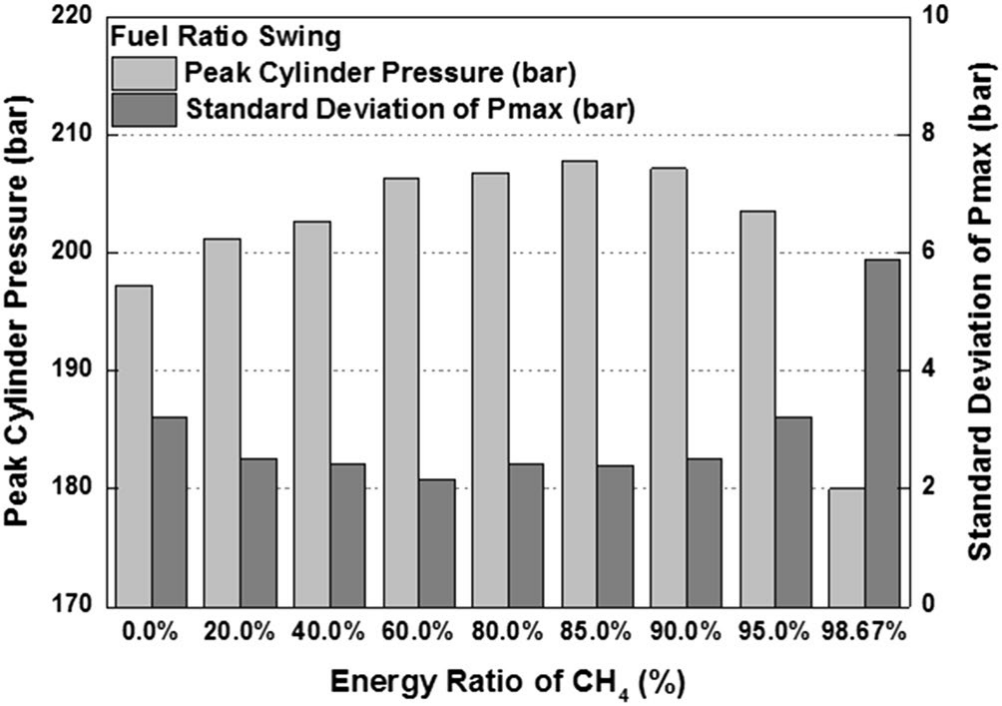
Peak cylinder pressure and combustion variation with respect to the fuel mixture ratio.

**Figure 11:**
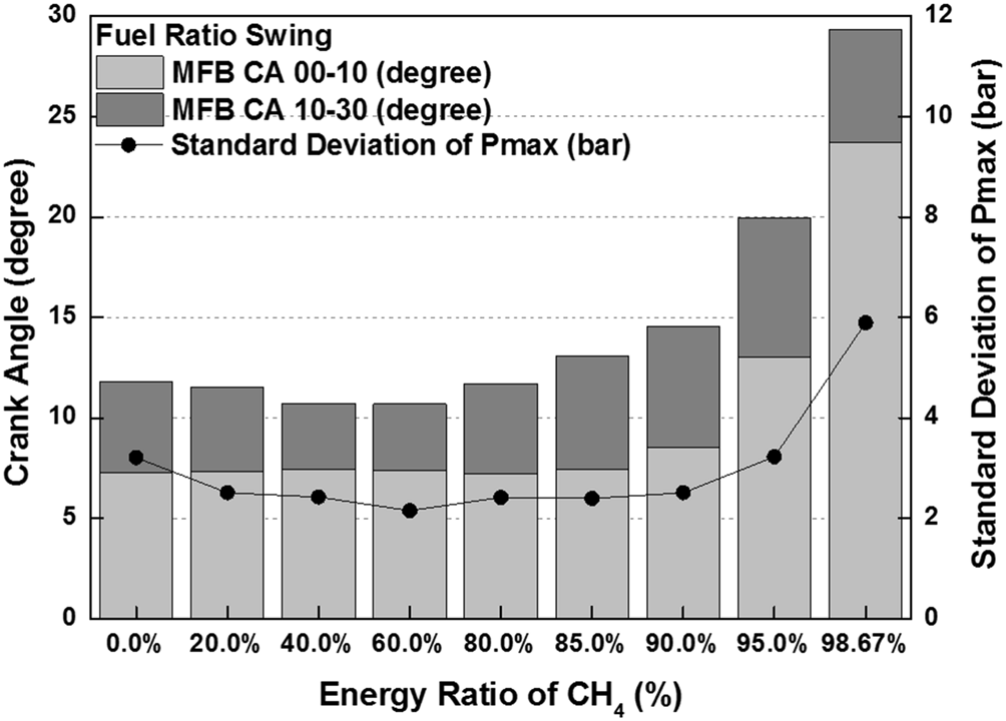
Relationship between MFB CA00-30 and combustion variation.

The cylinder pressure and rate of heat release (ROHR) with respect to the fuel mixture ratio are presented in Fig. 3. The cylinder pressure and ROHR, which shows the mixture form of diesel combustion and MPDF combustion, is determined by the mixture ratio of diesel and methane gas. As the methane mixture ratio increases, the main combustion timing is retarded and the peak cylinder pressure is reduced. After the main combustion duration, the combustion of residual gas in the cylinder is occurred, as a result, the ROHR is kept low regardless of the fuel mixture ratio. Moreover, it shows that the value of cylinder pressure of all experiment cases presents as almost the same at the end of combustion. This means that the low heating value of all experiment cases is consistent. The dual-fuel combustion is mixture form of diesel combustion and MPDF combustion. The following figures provide the detailed explanation for the form of diesel combustion and MPDF combustion. Figure 4 shows the ROHR of conventional diesel combustion. The pre-mixed combustion is occurred at the beginning of the combustion and ROHR is dramatically increased. After pre-mixed combustion, most of the diesel combustion consisted of rate-controlled and late combustion, and the ROHR remained lower than in pre-mixed combustion. As shown in Fig. 3, the injection of high quantities of diesel reflects the combustion form of diesel. With decreasing diesel injection ratio, on the other hand, pre-mixed gas combustion, which dramatically increased the ROHR by diesel combustion, was reduced. Rate-controlled combustion is also reduced. However, late combustion ROHR increased gradually, and as a result combustion forms converged in the form of MPDF combustion. In this study, the combustion forms of MPDF trend along with PREMIER combustion as shown in Fig. 5. According to C. Aksu et al.^24^, the MPDF combustion forms can be divided into ‘regular’, ‘PREMIER’ and ‘knocking’ combustion depending on engine load. The pre-mixed mixture ignition in the end-gas region where PREMIER combustion occurs, as noted by U. Azimov et al. ^25^, is located between regular and knocking combustion. PREMIER combustion consists of several combustion stages^26^. In the first stage, pilot injected diesel was ignited, and this increased ROHR slowly. Pre-mixed gas, however, was auto ignited in the end gas region at the second stage and this results in dramatically increased ROHR. During the third stage, ROHR was gradually decreased as the unburned residual gas slowly burned. For more detailed analysis, Fig. 6a,b shows the principles of diesel combustion and MPDF combustion, respectively. In the case of diesel combustion, the diesel fuel is used as the main source of the power. Therefore, the characteristics of combustion is dominantly affected by the low auto-ignition of diesel fuel. The injected diesel is mixed with the air and forms a homogeneous mixture in the combustion chamber during the ignition delay, and the high intensity of pre-mixed combustion is occurred. It can be a source of knocking combustion. After the pre-mixed combustion period, the diffusion combustion is dominantly shown, because high cylinder temperature reduced the ignition delay. Because the main sources of the NOx and PM emissions are the high temperature combustion and rich mixture, respectively^27,28^. In this period, while the region of locally stoichiometric equivalence ratio makes the NOx emissions, the PM emissions are formed form the locally rich mixture region. Unlike the diesel combustion, the most of pre-mixed gas fuel is used for the MPDF combustion. The diesel is only used as an ignitor. Form the ignition spot, the pre-mixed combustion is occurred and the flame surface is developed toward the cylinder wall. During the flame propagation, the auto-ignition is occurred from the locally high temperature region, causing the knocking combustion. Since the homogeneous mixture of MPDF combustion, the NOx and PM emissions are hardly produced. However, for the same reason, the low combustion temperature increases the incomplete combustion materials such as CO, THC and etc. The results of energy balances shown in Fig. 7 support the above explanation. Because of the high combustion temperature of diffusion combustion, the results of diesel 100% condition show the highest coolant, heat and exhaust losses, resulting the lowest fuel conversion efficiency. The more pre-mixed gas mixture ratio, the more reduced varieties of losses are observed. As a result, the fuel conversion efficiency of MPDF condition is the highest value. As well as the results of energy balances, the emissions results in accordance with the fuel mixture ratio can also be explained by the characteristics of diesel combustion and MPDF combustion. Figure 8 presents the normalized NOx, CO_2_ and THC emissions with respect to the fuel mixture ratio. The NOx emissions are exponentially reduced with the reduction of diesel mixture ratio, and this emissions trend demonstrates that the NOx emissions is primarily related with the combustion temperature. In the case of diesel combustion, the diffusion flame surfaces under the locally stoichiometric fuel–air mixture region forms the high temperature, and the NOx is produced in this region^29^. As decreasing the diesel mixture ratio, it is observed that reduction of the NOx emissions due to the low intensity of diffusion combustion. As a result, the NOx emissions are significantly reduced under the condition of the MPDF combustion. Unlike the trend of NOx emissions, the results of CO_2_ emissions represent that the linear relationship with the diesel mixture ratio. While the NOx emissions are closely related to combustion temperature, the CO_2_ emissions are proportional to the number of carbons in the fuel. Under the conditions of the same total low heating value of fuel, the diesel fuel has more carbons compared with methane gas, offering an explanation for the linear reduction in CO_2_ emissions correlating with decreased diesel fuel ratio. As decreasing the diesel mixture ratio, the THC emissions are exponentially reduced, that indicates the opposite trends with the NOx emissions. Contrary to the NOx generation principle, the THC emissions are ascribed to the low temperature combustion^27^. The higher diesel mixture ratio, the lower THC emissions are observed. However, increasing the pre-mixed gas mixture ratio, the misfiring is occurred in the locally lean fuel–air mixture region because of the low combustion temperature. As a result, the THC emissions are significantly increased. The combustion speed with respect to the fuel mixture ratio well explain the trends of NOx and THC emissions. Based on the ROHR, the timing of MFB is calculated to compare the combustion speed with respect to the fuel mixture ratio. Figure 9 presents the results of MFB with respect to the fuel mixture ratio. While the apparent difference of combustion speed with respect to the fuel mixture ratio is shown in CA 00–70, the timing of the CA90 is almost same regardless of the fuel mixture ratio. The MFB can be divided into two regions of main combustion and residual gas combustion. The main combustion duration is defined as the MFB CA 10 to CA 70. In this region, the remarkable variations of combustion speed could be observed with respect to the fuel mixture ratio. Until the methane gas mixture ratio between 0 and 80%, there was almost no difference in the combustion speed with respect to the fuel mixing ratio. However, over the condition of methane mixture ratio 80%, the combustion speed was dramatically reduced. As a result, while the combustion was started at bTDC 2.5 degree under the MPDF condition, in the other experimental conditions, the main combustion was almost finished. This result supports the trends of NOx and THC emissions with respect to the fuel mixture ratio. Because of the lowest combustion speed which causes the lowest combustion temperature in the MPDF combustion, the NOx is hardly produced. For the same reason, the MPDF combustion greatly increased THC emissions. After the MFB CA70, the residual gas combustion is occurred and this period shows the relatively slow and same combustion speed in all cases. In Fig. 9, the fastest combustion speed is presented in the condition of methane mixture ratio between 40 and 80%. This result is can be explained by the combination of diesel combustion and pre-mixed combustion. By decreasing the diesel mixture ratio, the proportion of diffusion combustion, which is located in the middle of the main combustion duration, was reduced. And flame surfaces could be spread rapidly from the wide range of ignition flame, instead. As a result, the initial pre-mixed combustion intensity and combustion speed are significantly increased. On the other hand, the lower combustion speed is observed under the diesel combustion and MPDF combustion. Under the high diesel mixture ratio, increasing the MFB CA50-70 can be explained by the diffusion combustion. Conversely, since the period of flame propagation from the ignition point is greatly increased with the lower diesel injection quantity, the MFB CA00-30 of MPDF combustion is the longest. During the combustion period MFB CA30-50, the high intensity of pre-mixed combustion is occurred in both diesel combustion and MPDF combustion. As a result, this period did not show a significant differences in all cases. The combustion duration with respect to the fuel mixture ratio affects not only the combustion pressure but also the combustion variation. Figure 10 shows the peak cylinder pressure and standard deviation of peak cylinder pressure with respect to the fuel mixture ratio. This figure shows the inversely proportional relationship between peak cylinder pressure and combustion duration. In contrast, the results of combustion variation with respect to the combustion duration indicate the opposite trend with the peak cylinder pressure. While the unstable and high combustion speed caused by the knocking combustion increases the combustion vibration, the results of this study shows that the combustion variation can be reduced by high dual-fuel combustion speed. In contrast, increasing the both diesel and methane gas mixture ratio make high combustion variation. In particular, the significantly high combustion variation is shown with the condition of MPDF. In order to analyze the effects of pre-mixed combustion on combustion variation, the relationship between the combustion period and combustion variation is compared. Figure 11 shows the relationship between the period of MFB CA00-30 and standard deviation of the peak cylinder pressure with respect to the fuel mixture ratio. This figure shows that the combustion variation is closely related to the initial pre-mix combustion period. During the initial pre-mix combustion period MFB CA00-30, the turbulent flow increases the flame propagation speed irregularly, resulting in the high combustion variation. According to the previous studies, unpredictable characteristics of ignition position and turbulent flow lead to increasing the combustion variation of SI combustion^30–32^. Similar to the SI combustion, as the diesel mixture ratio decreases, not only the turbulent flow but also the variation of ignition position is an important factor in increasing the combustion variation. Therefore, the turbulent flow and the variation of the ignition position are the main factors for increasing the combustion variation in the MPDF combustion during the early combustion period. For more detailed analysis, the optical combustion experiments were performed under the varieties of fuel mixture ratio by using the optical single cylinder engine.

### Optical engine experiment for the effects of fuel mixture ratio

**Figure 12:**
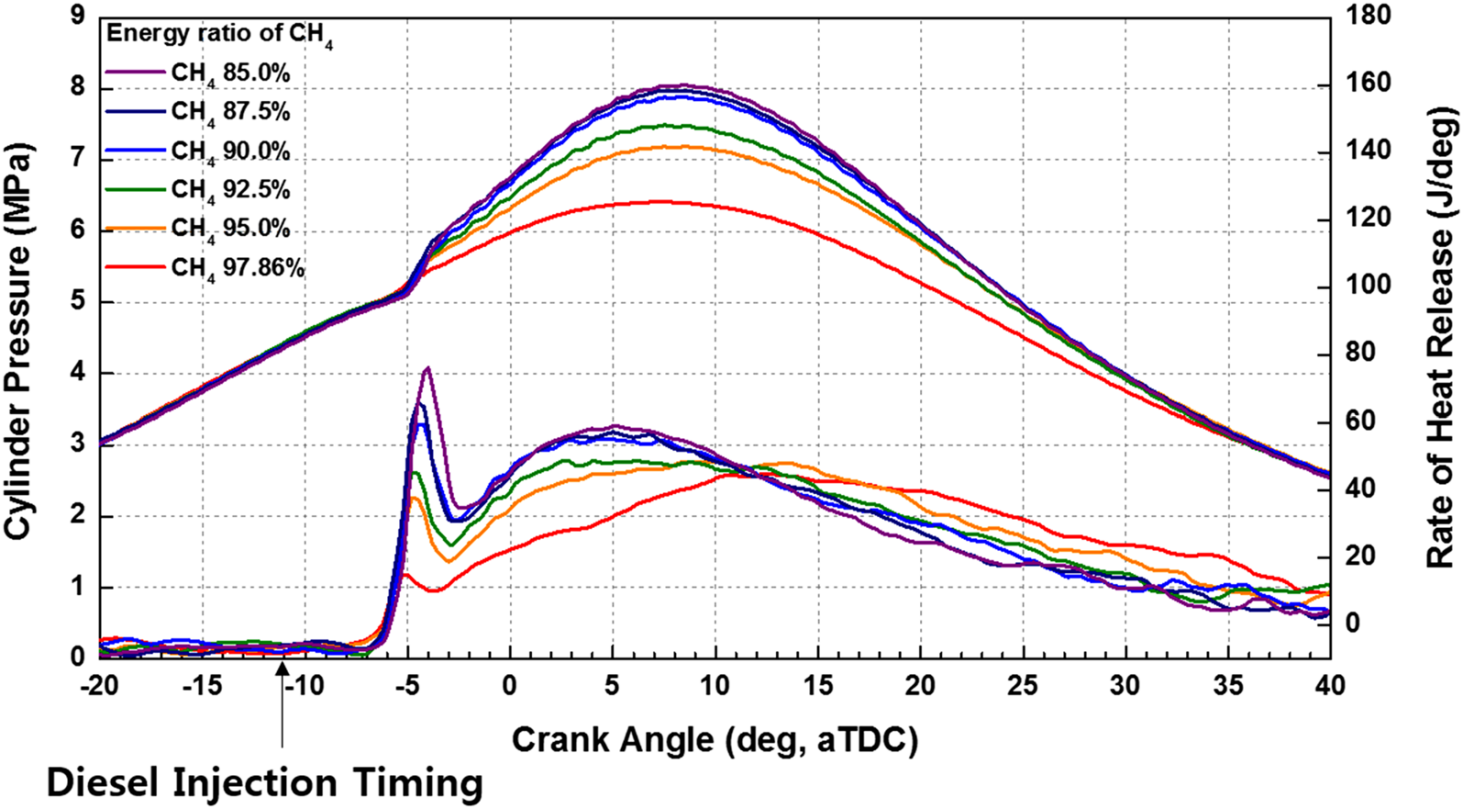
Cylinder pressure and ROHR with respect to the fuel mixture ratio in the optical engine.

**Figure 13:**
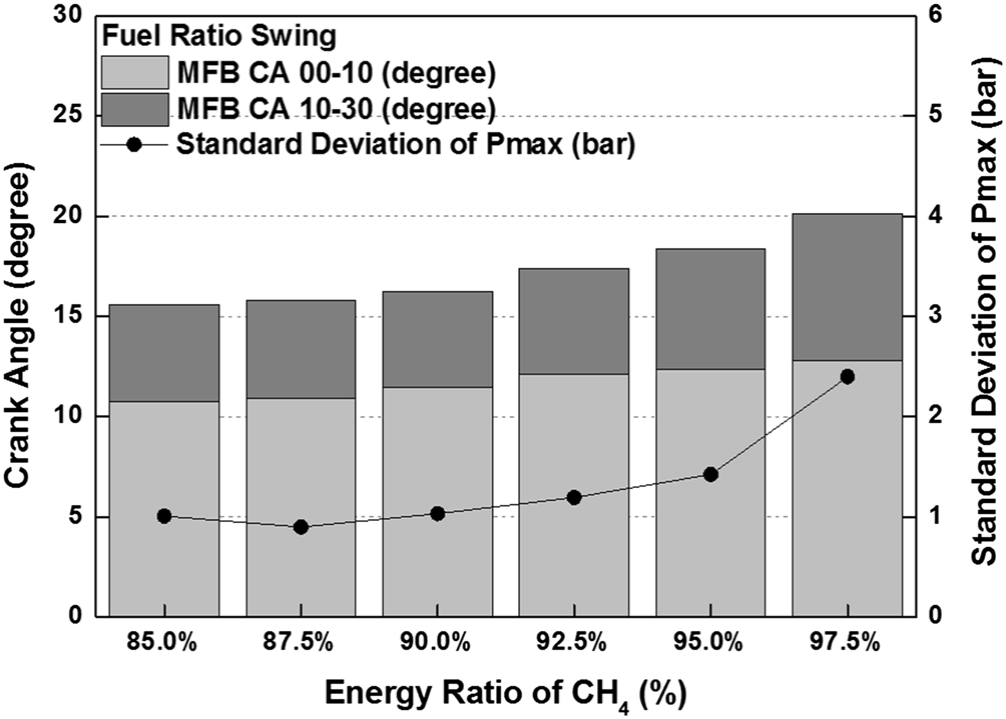
Relationship between MFB CA00-30 and combustion variation in the optical engine.

**Figure 14:**
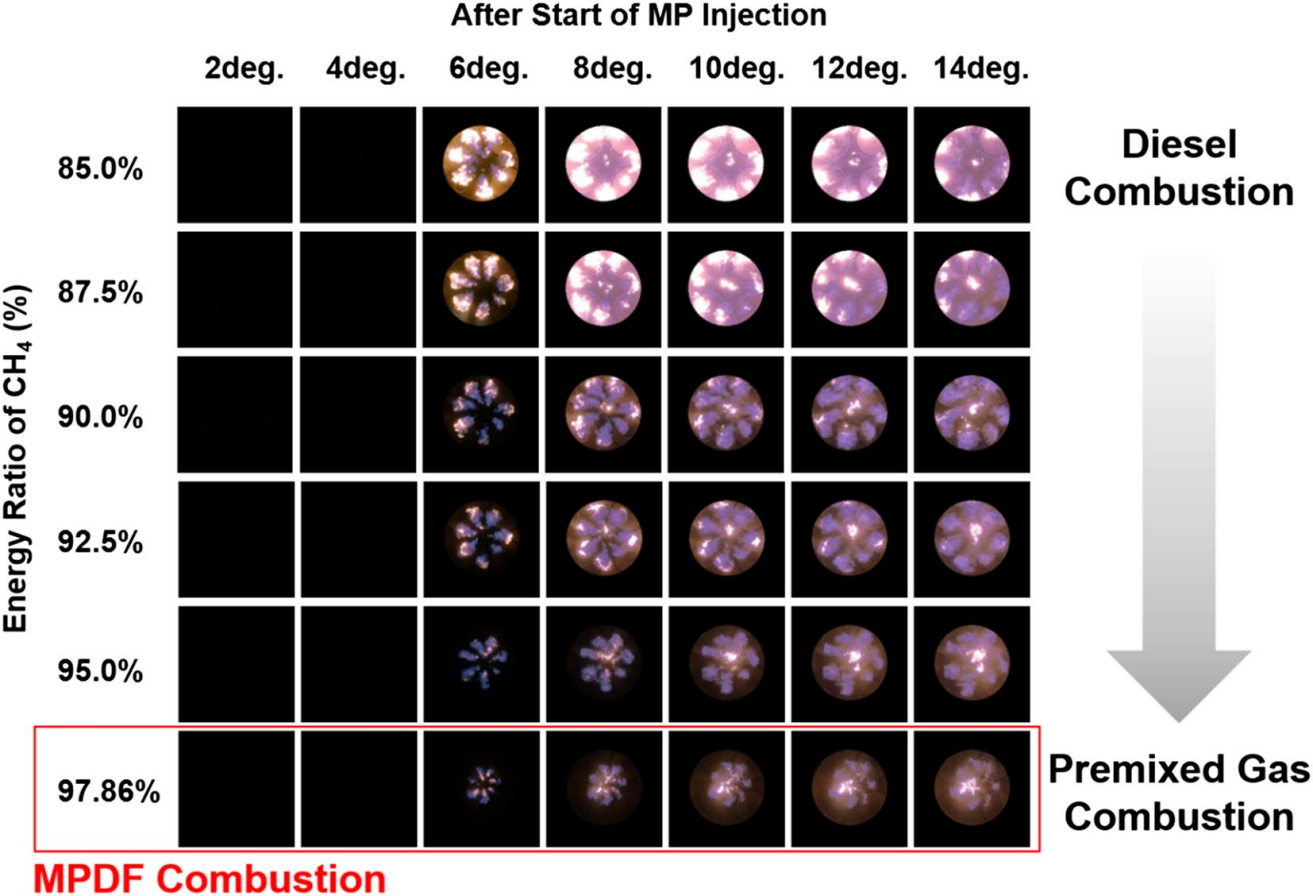
Combustion images depending on fuel mixture ratio.

**Figure 15:**
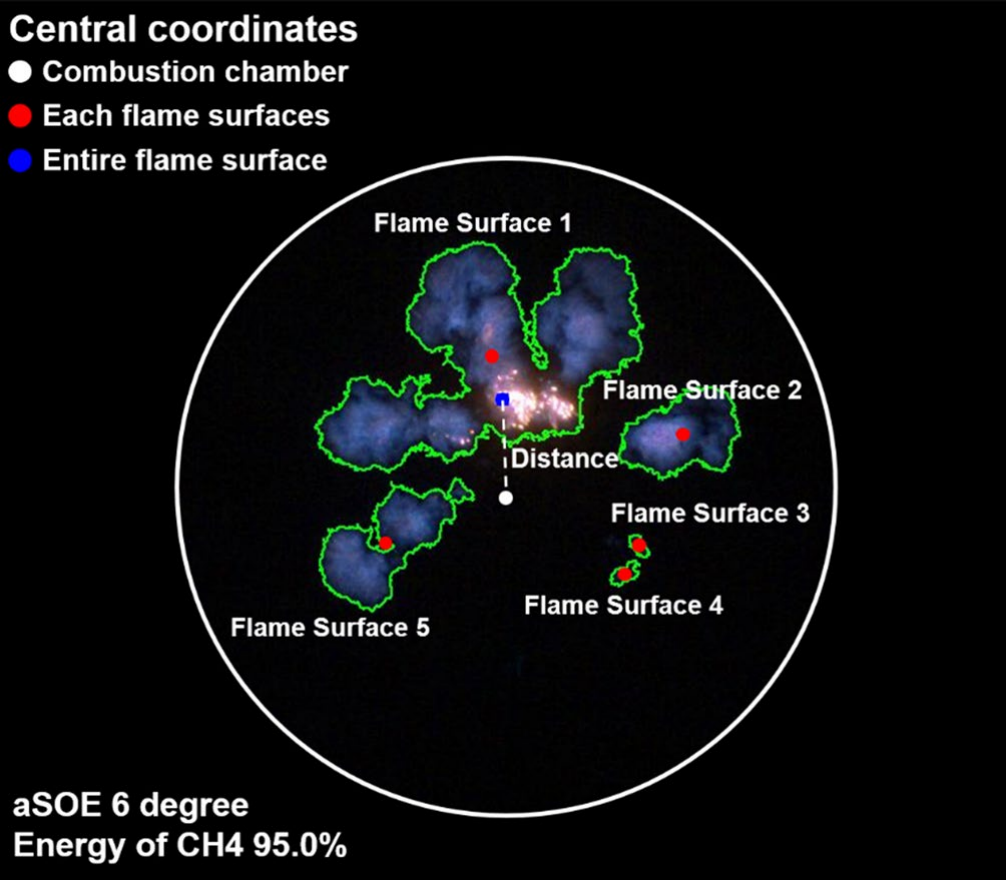
Central coordinates of combustion chamber, each flame surfaces and entire flame surface.

**Figure 16:**
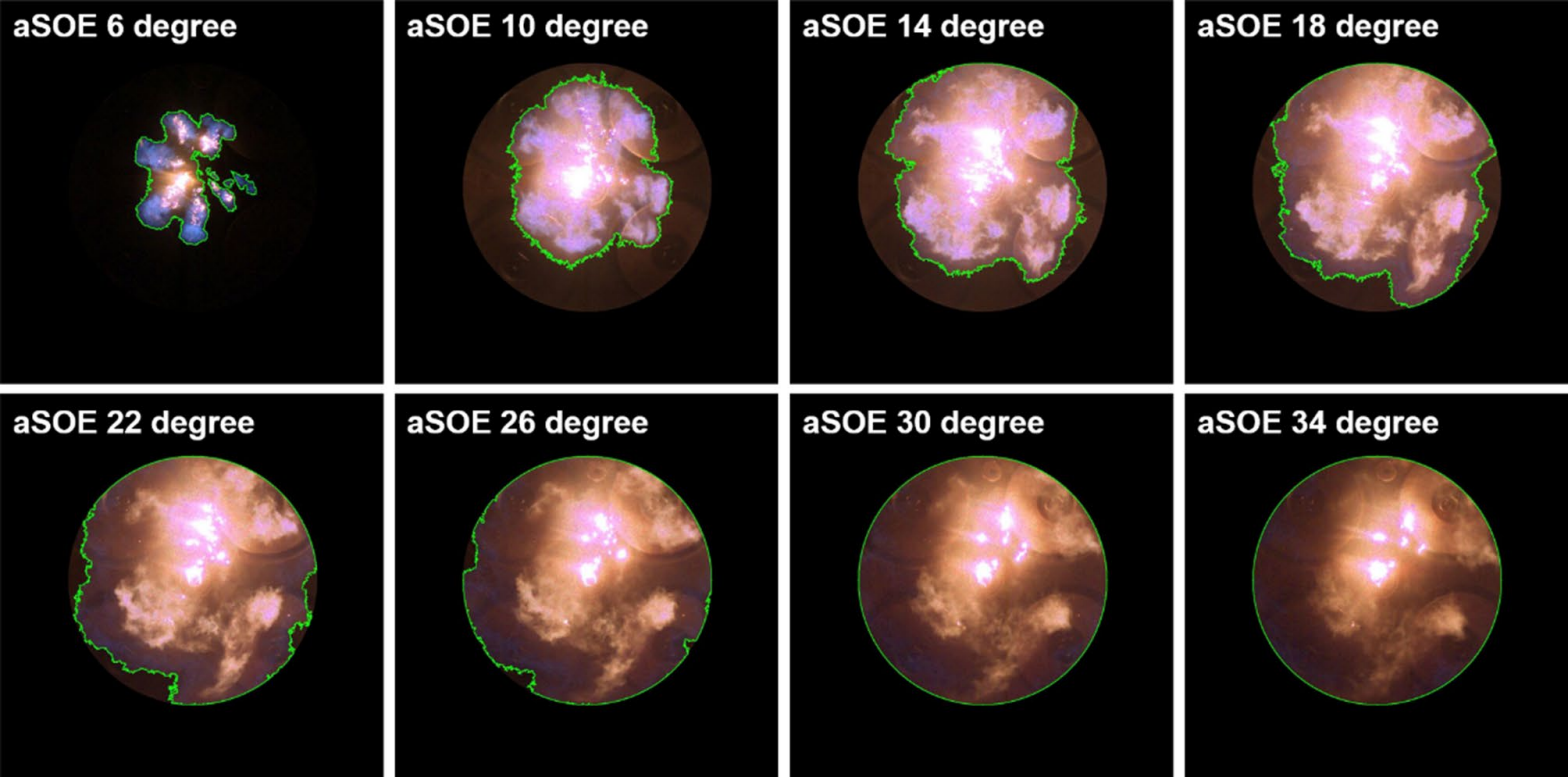
flame propagation process in the MPDF combustion.

**Figure 17:**
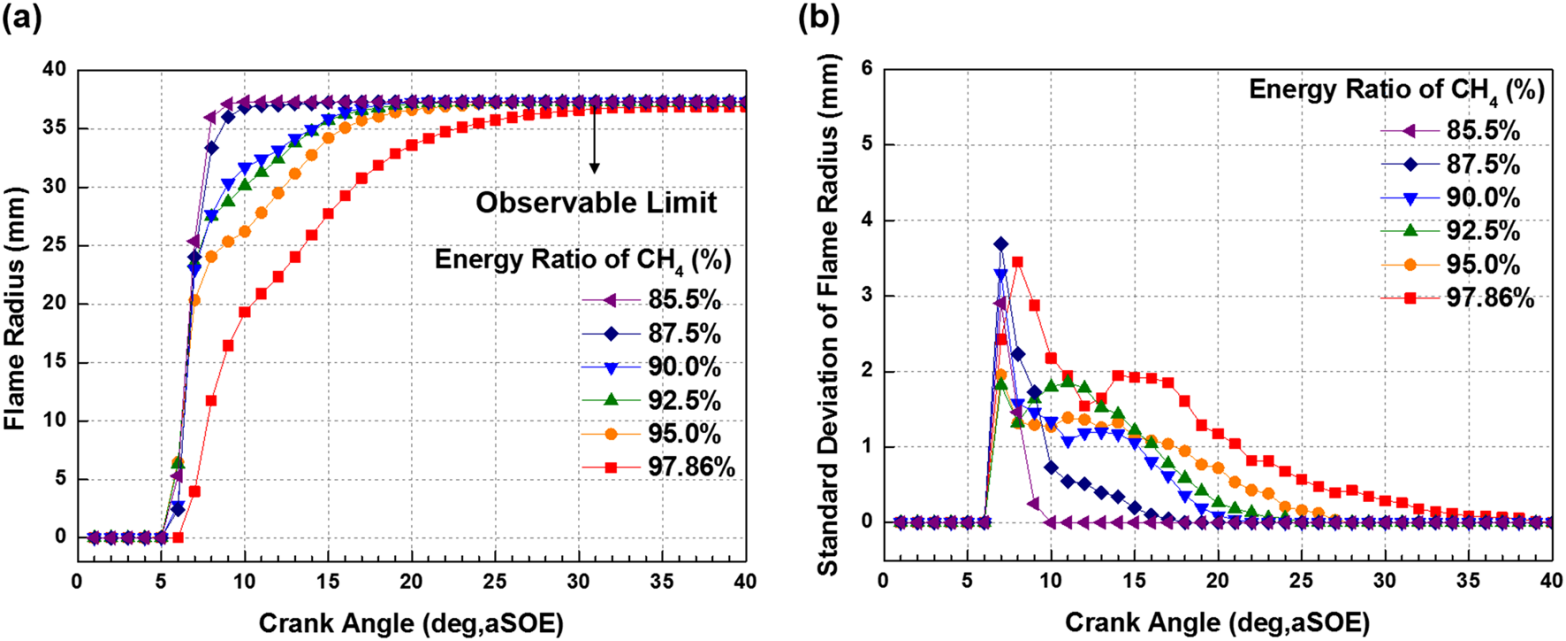
**(a)** Flame radius and **(b)** variation with respect to the fuel mixture ratio.

**Figure 18:**
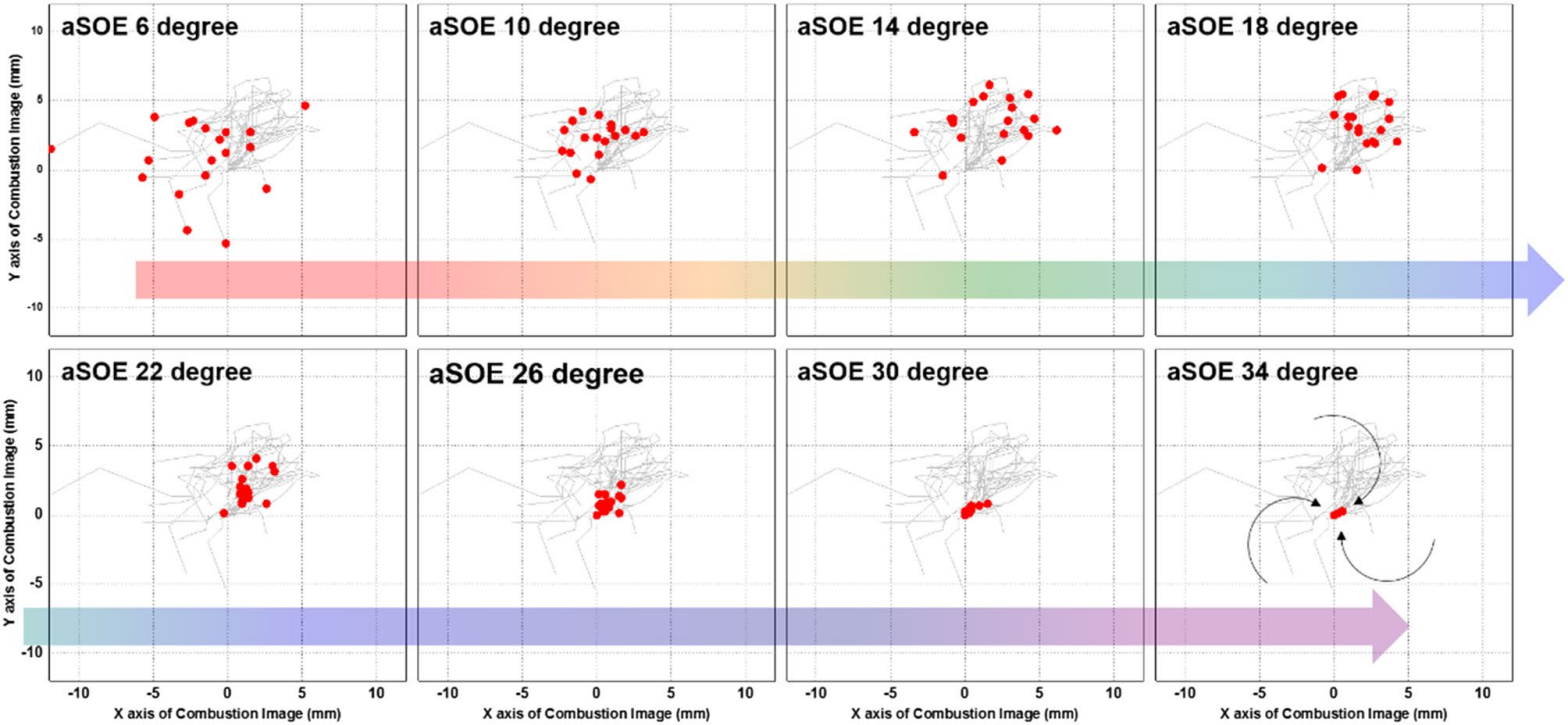
Variation of central coordinates of the flame surfaces in the MPDF combustion.

**Figure 19:**
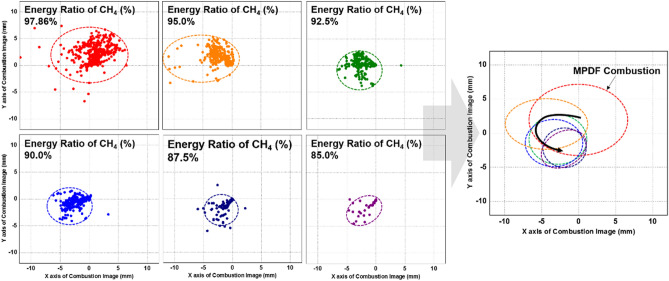
Variation of central coordinates of the flame surfaces with respect to the fuel mixture ratio.

**Figure 20:**
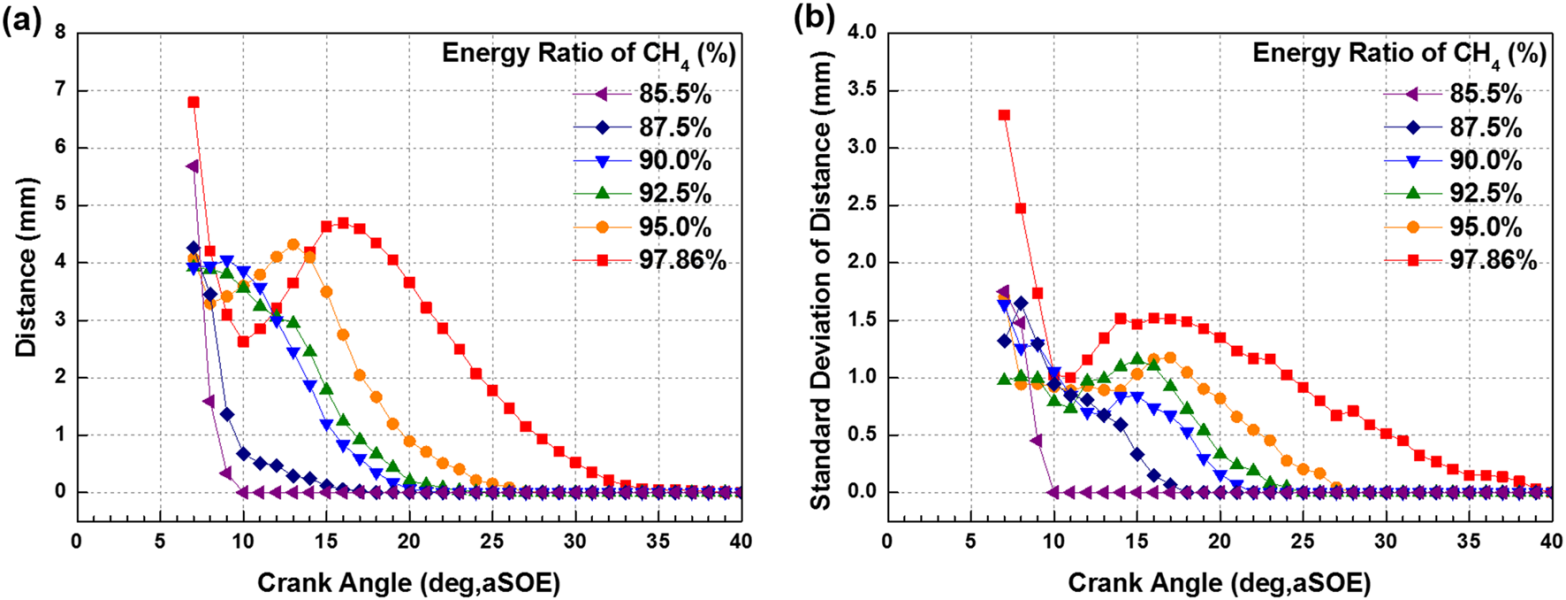
**(a)** Distance the center of between the combustion chamber and flame surfaces and **(b)** variation.

Figure 12 shows that the effects of fuel mixture ratio on the cylinder pressure and ROHR with respect to the crank angle. As shown earlier in metal engine results of Fig. 3, the less diesel mixture ratio makes lower the peak ROHR, which is caused by the pre-mixed combustion of diesel. As a results, the peak cylinder pressure which is proportional with the peak ROHR is reduced. The ROHR which represents the combustion form in the optical engine is different with that of the metal engine. Since the optical engine experiment was conducted under the relatively low engine load conditions than the metal engine experiment, the combustion form shown in Fig. 12 is the regular combustion. Under regular combustion conditions, the end-gas would not be auto-ignited, leading to no dramatic increasing in ROHR. Despite the differences in operating conditions of metal and optical engine, the MPDF combustion is still primarily affected by turbulent flow and the variation of ignition position. Figure 13 illustrates the relationship between the combustion variation and the duration of MFB CA00-30 in the optical engine. As shown in Figs. 11 and 13, the trend of experiments results in the optical engine are the same with that of the metal engine. With increased methane gas mixture ratio, both the combustion variation and the duration of CA00-30 are increased and this trend could be explained by unpredictable characteristics of ignition position and turbulent flow in the pre-mixed combustion. As well as the results of cylinder pressure, the combustion images are used to analyze the effects of turbulent flow and ignition positions on the combustion variation. Figure 14 shows the flame surface development with respect to the crank angle under the different fuel mixture ratio. During the ignition delay, the combustion flame could not be observed. The blue flame caused by pre-mixed combustion has been observed from the center of the combustion chamber in all experimental cases. In the MPDF combustion conditions, the blue flame is propagated from the center of the combustion chamber with the slowest speed. It means that the period affected by turbulence flow, which causes the combustion variation in the pre-mixed combustion, is increased with the higher pre-mixed gas mixture ratio. In contrast, the experimental results of increasing diesel mixture ratio indicate that instead of the blue flame, which is ascribed to the pre-mixed gas combustion, the spray plumes of the diesel show the diffusion combustion with its red flame. Even the condition of diesel mixture ratio 15%, most of the flame plumes shows the red color with not only the high brightness of the combustion images, and but also fast flame propagation speed. The combustion images with respect to the fuel mixture ratio well explain the trends of cylinder pressure and ROHR, moreover, it well be matched with the results of combustion speed and combustion variation. In particular, the combustion variation with respect to the fuel mixture ratio is obviously shown in the combustion images. The combustion image with the highest diesel mixture ratio shows that the diffusion flame already propagated out of the observable area at after SOE 6 degree. Additionally, there is less variation of the ignition position because of the higher diesel injection ratio. In contrast, the MPDF combustion images show both the lowest flame propagation speed and the highest variation of the ignition position. In order to analyze the effects of turbulent flow and the variation of ignition position on the combustion variation with respect to the fuel mixture ratio, the image processing was conducted on 20 images per crank angle. Through image processing using the MATLAB program, the boundary of the flame surface was distinguished and the central coordinates of the flame surfaces are calculated. Figure 15 shows the results of image processing. In this figure, the flame surfaces are divided into green border lines through image processing, and the white, red and blue dot indicate the central coordinates of the combustion chamber, each and entire flame surfaces, respectively. Through the image processing, as well as the average flame radius and the distance the center of between the combustion chamber and flame surfaces is calculated, and the effect of turbulent flow and the variation of ignition position with respect to the fuel mixture ratio can be analyzed. Figure 16 presents the images of flame propagation process under the condition of MPDF combustion. In the early combustion, each flames were propagated from the tip of the diesel plumes. After that, the flames surfaces were combined and developed. The combustion image of aSOE 14 degree shows that the flame surface has developed in one direction and some flame faces are already outside the visible area. It should be considered that these results cause errors when calculating the flame propagation speed and central coordinates of the flame. However, since the effects of these errors are not significant, and do not affect the experiments results in the early combustion, there is no problem with the analysis of the variation of the average flame radius and central coordinates of the flame surfaces. The average flame radius and cycle to cycle variation are shown in Fig. 17a,b, respectively. It shows that the higher gas mixture ratio, the slower the average flame radius reaches the observable limit, and the cycle to cycle variation is increased. In particular, the results of MPDF condition shows that the longest period for the average flame radius to reach the observable limit, the cycle to cycle variation of the average flame radius is the highest. Through this result, it is clearly found that increasing the methane gas mixture ratio increases the combustion variation because of the higher effects of turbulent flow on the pre-mixed combustion. As well as the turbulent flow, the effects of variation of ignition position on the combustion variation can be confirmed through the results of central coordinates of flame surfaces with respect to the fuel mixture ratio. Figure 18 presents center of flame surfaces in the MPDF combustion with respect to the crank angle, and one point corresponds to one cycle. At the beginning of combustion, the ignition position is widely spread, but the points of center of the flame surfaces are converged into the center of the combustion chamber with the developing flame surfaces. Each line is the trails of central coordinates of the flame surfaces, and it is converged to the direction of clockwise which is the direction of turbulent flow in the combustion chamber. In addition to the condition of MPDF, the central coordinates of the flame surfaces were calculated for all experimental conditions, and it is shown in Fig. 19. In the MPDF condition, the points which means the central coordinates of flame surfaces are most widely distributed, however, as the diesel mixture ratio increases, the points are converged to the center of the combustion chamber with the direction of counter clockwise. Figure 20 quantitatively presents the results of Fig. 19. Figure 20a,b indicate the average distance and variation between the center of flame surfaces and the center of combustion chamber, respectively. The both values are significantly reduced by increasing the diesel mixture ratio. This figure demonstrates that increasing the diesel mixture ratio lower the combustion variation by reducing the variation of ignition position. Through the optical experiment, it can be explained why the combustion variation in MPDF combustion was increased compared with the other experiment conditions.

## Conclusion

In this study, the characteristics of MPDF combustion with respect to the fuel mixture ratio were investigated in a heavy duty single cylinder metal and optical dual-fuel engine. The results of this study are summarized as follows.

1. In the dual-fuel combustion, the combustion phase is determined by fuel mixture ratio. If the diesel mixture ratio was increased, the diesel combustion became the dominant combustion form. Higher methane gas ratio, on the other hand, the dual-fuel combustion form is changed to the MPDF combustion. In consideration of metal engine experiment, the PREMIER phase of MPDF combustion was indicated. This phase is located between regular and knocking combustion.
2. Increasing the methane mixture ratio improves the fuel conversion efficiency about 5% by reducing the coolant, heat ant exhaust losses. As well as the fuel conversion efficiency, the MPDF condition effectively reduced the NOx emissions about 90%p. However, the THC emissions are increased about 95%p with the MPDF combustion. The NOx and THC emissions are closely related to the combustion temperature. The locally high combustion temperature region increased the NOx emissions with the diffusion combustion, in contrast, the THC emissions are ascribed to the pre-mixed combustion due to the lower combustion temperature. The CO_2_ emissions were found to be proportional to the number of carbons in the fuel and the experimental results of 100% diesel mixture ratio shows the highest CO_2_ emissions.
3. While the results of methane mixture ratio between 40 and 80% show the lowest combustion variation, the highest combustion variation is observed under the MPDF combustion. Through the optical engine experiment, it can be found that the cycle to cycle combustion variation is ascribed to the turbulent flow and the variation of ignition position. In the early combustion period, the turbulent flow increases the combustion variation by increasing the uncertainty of flame propagation during the pre-mixed combustion.

## Data Availability

The data that support the findings (experimental results) of this study are available from the corresponding author upon reasonable request.

## Abbreviations

aSOE: After start of energizing
aTDC: After top dead center
bTDC: Before top dead center
CO: Carbon monoxide
CO_2_: Carbon dioxide
CH_4_: Methane
DF: Dual-fuel
IMEP: Indicated mean effective pressure
MPDF: Micro-pilot dual-fuel
NOx: Nitrogen oxides
PM: Particulate matter
PREMIER: The remixed mixture ignition on the end-gas region
SOE: Start of energizing
SI: Spark ignition
THC: Total hydrocarbon
